# Genomic signatures of natural selection at phenology-related genes in a widely distributed tree species *Fagus sylvatica* L

**DOI:** 10.1186/s12864-021-07907-5

**Published:** 2021-07-31

**Authors:** Joanna Meger, Bartosz Ulaszewski, Jaroslaw Burczyk

**Affiliations:** Department of Genetics, Faculty of Biological Sciences, Kazimierz Wielki University, Chodkiewicza 30, 85-064 Bydgoszcz, Poland

**Keywords:** Forest tree, Genotype-environment association, Local adaptation, Sequence capture, Candidate genes, Bud-burst phenology

## Abstract

**Background:**

Diversity among phenology-related genes is predicted to be a contributing factor in local adaptations seen in widely distributed plant species that grow in climatically variable geographic areas, such as forest trees. European beech (*Fagus sylvatica* L.) is widespread, and is one of the most important broadleaved tree species in Europe; however, its potential for adaptation to climate change is a matter of uncertainty, and little is known about the molecular basis of climate change-relevant traits like bud burst.

**Results:**

We explored single nucleotide polymorphisms (SNP) at candidate genes related to bud burst in beech individuals sampled across 47 populations from Europe. SNP diversity was monitored for 380 candidate genes using a sequence capture approach, providing 2909 unlinked SNP loci. We used two complementary analytical methods to find loci significantly associated with geographic variables, climatic variables (expressed as principal components), or phenotypic variables (spring and autumn phenology, height, survival). Redundancy analysis (RDA) was used to detect candidate markers across two spatial scales (entire study area and within subregions). We revealed 201 candidate SNPs at the broadest scale, 53.2% of which were associated with phenotypic variables. Additive polygenic scores, which provide a measure of the cumulative signal across significant candidate SNPs, were correlated with a climate variable (first principal component, PC1) related to temperature and precipitation availability, and spring phenology. However, different genotype-environment associations were identified within Southeastern Europe as compared to the entire geographic range of European beech.

**Conclusions:**

Environmental conditions play important roles as drivers of genetic diversity of phenology-related genes that could influence local adaptation in European beech. Selection in beech favors genotypes with earlier bud burst under warmer and wetter habitats within its range; however, selection pressures may differ across spatial scales.

**Supplementary Information:**

The online version contains supplementary material available at 10.1186/s12864-021-07907-5.

## Background

Local adaptation is one of the most important evolutionary mechanisms allowing species to thrive across heterogeneous environments [1]. Populations are said to be locally adapted when individuals from resident populations have higher fitness than individuals of the same species introduced from other habitats [2]. Knowledge about the extent of local adaptation and its underlying mechanisms in natural populations provides the basis for predicting responses to environmental fluctuations, including those associated with global climate change.

The genetic underpinnings of local adaptation, though, are poorly understood. Local adaptation would be expected to change allelic frequencies of genes affecting fitness in particular environments. However, most phenotypic traits related to adaptation are typically quantitative polygenic traits, which complicates the identification of genetic polymorphisms linked to adaptation [3, 4]. Local adaptation has been considered an important factor in maintaining genetic variation within species, but environmental heterogeneity also favors the evolution of adaptive phenotypic plasticity [1]. However, because phenotypic plasticity does not necessarily have to be adaptive [5], its interplay with adaptive traits confounds the ability to find genetic determinants of adaptation.

Local adaptation is best studied in long-lived sessile organisms with large effective population sizes spanning large, variable environments [6], such as forest trees [7]. In plants generally, the extent of any local adaptation can be assessed based on reciprocal common-garden experiments; however, phenotypic and genetic differentiation along native environmental gradients can also provide some evidence for local adaptation [3, 8]. Studying local adaptation in forest trees has been of interest for researchers for a long time, which is emphasized in many common-garden experiments established for various tree species, motivated mainly by the management of forest reproductive material and tree improvement programs. Such experimental sites are nowadays excellent platforms for studying local adaptation [9], allowing for the exploration of complex relationships between phenotype, genotype, and environment at the genomic level [4, 10]. Common-garden trials have the potential to provide the links between GPA (genotype-phenotype association) and GEA (genotype-environment association) studies [11], since the genomic diversity of populations can be associated to environmental variables existing at the place of the origin of each population. However, at the same time, this genomic diversity can be associated with phenotypes considered as a population response to environmental conditions existing at an experimental site.

Phenology of tree development is one example of a set of traits thought to be strongly indicative of adaptive fitness. Local population adaptations have been described among tree species for the timing of bud burst in relation to local climatic conditions varying along latitude or altitude [12–14]. As an example of a connection to fitness, bud burst phenology is impacted by the length of the growing season, and it has consequences for biomass production. Earlier bud burst and later bud set extend the growing season and increase net photosynthetic productivity, thus increasing an individual’s competitive ability [14, 15]. On the other hand, later bud burst and earlier bud set improve cold resistance [16]. Bud burst timing also regulates biological interactions between trees and associated species (pest insects, pathogens, mycorrhizal fungi), and phenological changes usually result in different synchrony with insect herbivores and fungal pathogens, which also leads to consequences for overall fitness [17, 18].

Modifications of the phenology of flowering, bud burst, or bud set have been observed in relation to global climate changes [19, 20]; however, the response of any individual tree species is hard to predict. While some authors suggest that a warming climate may promote earlier bud flashing with an associated extension to the growing season [15], others highlight the importance of photoperiod and of chilling requirements in some species, along with problems related to increased aridity of the environment [21]. The chance for late frost damage may increase due to the overall shift in bud burst dates [15, 22]. Nevertheless, observations of high phenological plasticity in forest trees suggests the potential for rapid population responses to variations in temperature [23–25].

The genetic background of bud burst phenology has been investigated in a number of tree species [26, 27]; see also review in Howe et al. [16]. In general, bud burst was found to be under strong genetic control with high heritability [16], increasing the prospects of identifying genomic regions controlling this trait. However, in oak (*Quercus robur*), it has been estimated that bud burst phenology, being considered a typical quantitative trait, is controlled by at least 12 unique genes or chromosomal regions [28]. If this complexity is representative of other species, it will mean that discoveries of genomic backgrounds to phenology will be complex. Indeed, functional genomics of phenology may actually be far more complicated than even this, as different genomic regions might play different roles in different populations subjected to different environmental pressures [25, 26, 29], which may be further confounded by possible epigenetic effects [30].

Next-generation sequencing approaches have provided a new handle on the genetic basis of such complex adaptive traits at whole-population levels [31–33]. Among these traits, phenology appears to be of primary interest in the literature. Attempts have been made to identify candidate genes responsible for bud burst phenology in the woody plants *Vitis* [34] and *Ribes* [35], but one of the first studies was performed on the tree species *Quercus* [36]. Derory et al. [36] identified approximately 190 genes down- or up-regulated during bud burst in sessile oak, which were therefore annotated as potential sources of signatures of selection. In a subsequent study, nine candidate genes were assessed for bud burst timing association [37]. Patterns of diversity among natural and segregating populations were, however, variable, making final inferences on the significance of respective genes difficult.

Other approaches have also been tested. Although genomic scans became attractive tools for studying genomic diversity in trees [38, 39], their applicability to the study of genomic signatures of adaptation has been debatable [8, 40, 41]. Instead, SNP arrays, exome, or whole-genome sequencing have been more suitable tools with which to conduct detailed identification [7, 42, 43]. Finally, several authors pointed out the necessity of studying intergenic allelic associations in candidate genes across a wide range of environments to test intergenic disequilibria and multilocus differentiation measures [29, 37].

Among the typical model tree species used for such studies, European beech (*Fagus sylvatica* L.) is widespread, and is one of the most important broadleaved trees in Europe. It has high importance not only economically, but also ecologically, being the dominant tree species in many forest ecosystems [44, 45]. As a late-successional tree species, *F. sylvatica* might be considered the perfect model species for studying local adaptation because of its importance as a component in its metapopulation dynamics.

The adaptation potential of beech to climate change has been widely discussed. While beech is considered a sensitive tree species in the context of predicted environmental changes, some authors conclude that beech will not lose its importance and adaptedness in the future [46–51]. However, changes in marginal beech populations have already been observed [52, 53], and different modeling studies predict range shifts for this species in the context of global warming [54, 55]. Therefore, a deeper understanding of its potential for adaptation to changing environmental conditions is required.

Beech bud burst phenology has been investigated thoroughly, and its adaptive importance is well-documented [56–61]. Recently, multiple SNP markers have been described in climate-related candidate genes in European beech or other Fagaceae [36, 62–67]. These SNPs have been successfully used to detect genetic variation showing signatures of selection in beech [68–70].

Detecting such signatures of selection is not a trivial task. In the last decade, several analytical methods have been developed to detect putative adaptive loci from genomic datasets, making it possible to assess adaptive genetic variation in natural populations [71–73]. Genotype-environment association analyses (GEA) are particularly promising for detecting these loci [72]. Unlike differentiation outlier methods, which identify loci with strong allele frequency differences among populations, GEA approaches directly associate allele frequencies and environmental conditions not only to detect genetic variants putatively under selection, but also to characterize the environmental conditions contributing to adaptive genetic variation [10, 71, 72, 74]. GEAs are especially promising because they are better able to detect relatively weak signals of selection compared to methods based on population differentiation [75–77]. In particular, multivariate GEA methods (which analyze many loci and environmental predictors simultaneously) identify how groups of loci covary in response to environmental predictors, and may reduce the need for multiple testing while potentially identifying polygenic selection [72, 78]. This is important because many adaptive processes are expected to result in weak multilocus signatures due to selection on standing genetic variation which has not yet led to allele fixation [3, 79, 80].

Environmental association studies such as these have been conducted in a variety of organisms, including forest trees. For instance, associations of genetic variation with temperature and precipitation have been detected in *Alnus glutinosa* [81], *Populus balsamifera* [82], *Populus trichocarpa* [83], *Pseudotsuga menziesii* [84], *Quercus lobata* [85], *Quercus rugosa* [86], *Picea abies* [87], and *Pinus taeda* [33]. More recently, SNPs in candidate genes that may be under climate selection have been found in European beech [63, 66–69, 88], and associations between these SNPs and environmental variables such as temperature, precipitation, and drought have been determined.

Less is known about the precise molecular basis of phenology-related traits; however, genomic resources facilitating such research in beech have been growing only recently. For instance, Lesur et al. [89] revealed a large set of genes involved in dormancy regulation in beech, and Mishra et al. [90] developed the reference genome for European beech, allowing the identification of SNP polymorphisms within well-defined gene models. Lalagüe et al. [63] and Müller et al. [65, 91] also analyzed a selection of candidate genes related to bud burst in European beech. While these studies are valuable, the field is nascent, and studies directly investigating relationships between genetic markers and phenology in European beech are scarce.

Here, we use a candidate gene approach to detect genotype-environment (GEA) and genotype-phenotype (GPA) associations to look further for genomic signatures of local adaptation of European beech to climate heterogeneity across Europe. Unlike genome-wide studies, candidate gene techniques focus on genes of a priori interest, and therefore are expected to enrich for the number of significant associations (e.g. [92]).

We focused on candidate genes differentially expressed during bud burst in European beech [89]. Based on the sequence capture approach, we generated a SNP dataset to: (i) detect a spatial pattern of genetic variation (geographic variables); (ii) identify SNPs associated with climatic variables existing at the original locations of populations; and (iii) find relationships between genetic polymorphisms and the subset of adaptive traits (such as spring and autumn phenology scores, survival rate, and tree height) measured at the location of a common-garden trial. We hypothesize that, given the variable pattern of climatic variables across the species’ range, the strength of selection will be variable in different parts of the species distribution. It is expected that, because we selected candidate genes related in some way to bud dormancy and its release, several identified SNPs will demonstrate associations to climate and phenology-related phenotypic variables.

## Results

### Environmental variables

**Fig. 1:**
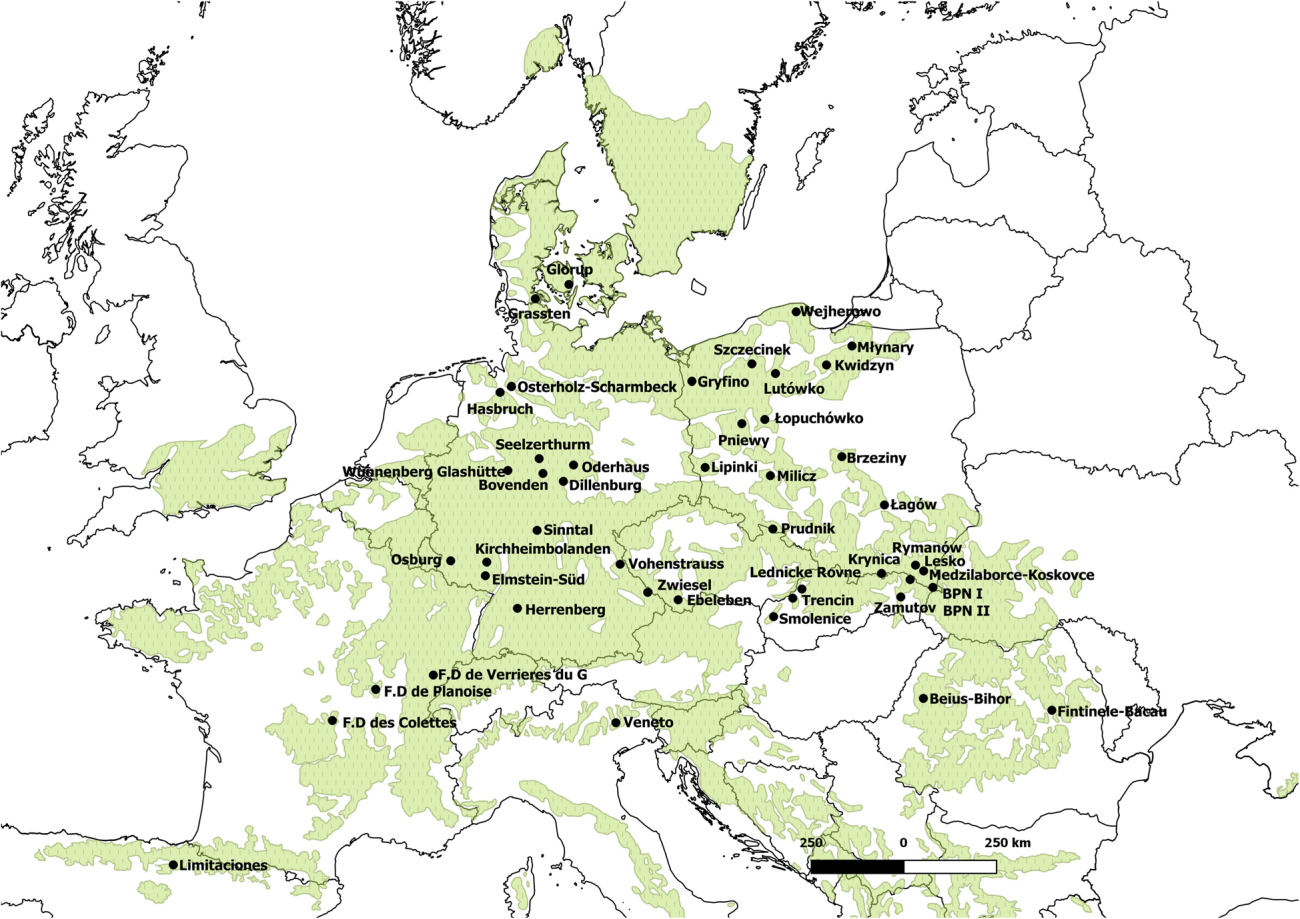
Geographical distribution of the sampled provenances in Europe (black dots) and natural distribution of *Fagus sylvatica* (green)

**Fig. 2:**
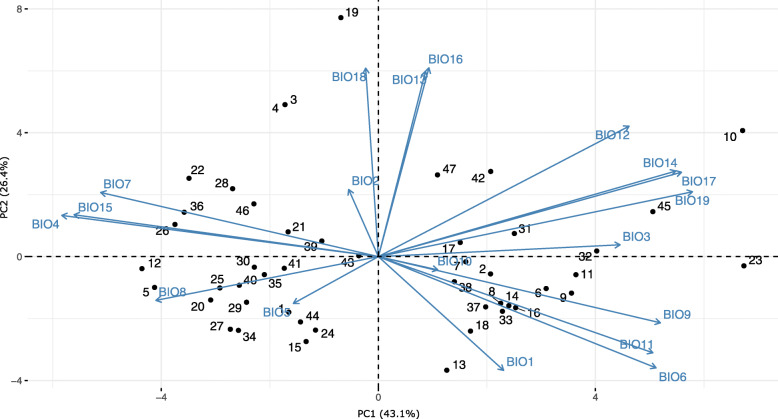
PCA plot based on 19 environmental variables (BIO1-BIO19) describing 47 European beech sampling sites

Principal component analysis revealed that much of the variation in environmental variables observed at the locations of sampled populations (Fig. 1) could be explained by the first three principal components (PC1, PC2, and PC3), which explained 85.21% of the total variance of the environmental variables data and were chosen as climate variables in further analyses. These climate indices describe the main spatial features of the climate in Europe (Table S3). In PC1 (43.05% of the variance), minimum temperature in the coldest month (BIO6), mean temperature in the driest quarter (BIO9), mean temperature in the coldest quarter (BIO11), precipitation in the driest month (BIO14), precipitation in the driest quarter (BIO17), and precipitation in the coldest quarter (BIO19) were all strong positive correlates, with loadings greater than 0.80. However, temperature seasonality (BIO4), annual temperature range (BIO7), and precipitation seasonality (BIO15) were the strongest negative correlates, with loadings under − 0.80. For PC2 (26.45% of the variance), precipitation in the wettest month (BIO13), precipitation in the wettest quarter (BIO16), and precipitation in the warmest quarter (BIO18) showed loading over 0.80. In PC3 (15.71% of variance), only mean diurnal range (BIO2) and maximum temperature in the warmest month (BIO5) were highly positively correlated (*r* > 0.80; *p* < 0.001). The biplot constructed by the two principal components showing populations and 19 environmental factors (as vectors) is presented in Fig. 2. The first principal component (PC1) separated Western European from Eastern European populations, which were characterized by a higher temperature in the coldest month (BIO6) and the driest quarter (BIO9), and also higher rainfall in the driest (BIO17) and coldest (BIO19) quarters. Along PC2, the Southern European populations occupying habitats that were characterized by greater rainfall in wettest (BIO16) and warmest (BIO18) quarters were separated from the remaining populations.

In general, the climate variables defined by PCs were significantly related to geographic variables, as indicated by canonical correlation analysis (*Can R* = 0.955; *p* < 0.0001; Table S4). Specifically, PC1 was significantly correlated with longitude (*r* = − 0.908, *p* < 0.001), PC2 had the strongest correlation with altitude (*r* = − 0.720; *p* < 0.001), and PC3 was strongly correlated with latitude (*r* = − 0.693; *p* < 0.001) (Table S4). The distribution of PC scores across provenance locations is presented in Figure S1. PC1 seems to represent the gradient from Atlantic to continental climate (mostly west-east direction), while PC2 and PC3 are more related to environmental variables varying along the north-south direction.

### Phenotypic variables

Canonical correlation analyses, in general, indicated no relationship between phenotypic measures and climatic or geographic variables (Table S4). Only survival was found to be moderately correlated with PC3 (*r* = 0.411; *p* = 0.004). It should be noted that survival was also correlated with tree height and autumn phenology (*r* = 0,456; *p* = 0,001 and *r* = 0.343; *p* = 0,018) (Table S4). Based on spring (*S*) and autumn (*A*) phenology scores we calculated a synthetic variable (*L* = *S* - *A*), which is intended to mimic the length of the growing season. Provenances with the earliest growth in the spring and the latest growth cessation in autumn have the highest values of *L*. We found that this variable was strongly correlated with survival (*r* = 0.445; *p* = 0.002), but not with tree height (*r* = 0.227; *p* = 0.125).

### Sequence capture data

An average of 5,636,543 raw sequence reads was generated per individual tree. More than 99% of reads were mapped to the reference genome of European beech (Mishra et al. 2018). Any sequence reads aligned with unique positions were subjected to SNP calling across individuals, identifying 15,742 SNPs (unfiltered). After applying the filtering criteria, as described in *Material and methods*, before LD pruning there were still 11,307 SNPs; however, after LD pruning a total of 5970 polymorphic SNPs were retained and used in further analyses.

From the initial set of 485 candidate genes used in the sequence capture experiment, and based on the reference genome [91], we identified 380 well-defined gene models, including 277 complete and 103 partially valid (without UTR sequences) gene models. Cumulative sequence length was equal to 1,014,286 bp (Table S5). The majority of gene models were supported by existing gene structure annotations of *F. sylvatica*. However, we found some annotation errors. In some cases, different initial contigs collapsed into the same gene model, or some initial candidate genes could not be identified in the *F. sylvatica* genome, likely due to gaps in the reference assembly.

We used only 2909 SNPs located within, or up to 500 bp from, 380 validated gene models for further analyses. By position relative to gene models, 2554 SNPs (87.79%) were located within candidate genes, with an average SNP density of 2.5 SNPs/kbp (or one SNP per 400 bp). 355 SNPs (12.21%) were found within a distance of 500 bp from gene models. Based on gene model annotations, 1889 (64.93%) were located in exons, 1511 (51.94%) in coding regions (CDS); 240 (8.25%) in 5′ untranslated regions (5′ UTRs), 138 (4.74%) in in three prime untranslated regions (3′ UTRs), and finally 665 (22.86%) in introns (Table 1).

**Table 1:** Number and percent of 2909 SNPs located in different genomic regions

Category	No. of SNPs	%
**Exon**	1889	64.94
**CDS**	1511	51.94
**5′ UTR**	240	8.25
**3′ UTR**	138	4.74
**Intron**	665	22.86
**Unclassified**	355	12.20

The level of missing data was low because only 3 to 44 loci per individual were missing (an average of 20.39 SNPs per individual; 0.75%), and 0 to 4 individuals out of 92 had a missing genotype within a locus (an average of 0.723 individuals per locus; 0.75%). We identified only one example of copy number variation (CNV). This was found in gene FSB010001901 (GDSL esterase/lipase) that was either gained (4 cases) and lost (88 cases) among the 92 individuals.

### Genetic diversity and structure

The mean genetic diversity of the 2909 SNP loci for the whole sampe was found to be 0.29, indicating a relatively high level of genetic diversity. An analysis of structure revealed the most likely number of genetic clusters as *K* = 3 (Figure S2), the first (K1) containing 39 individuals (present predominantly in Southeastern Europe), the second (K2) comprising 30 individuals (present predominantly in Western Europe), and the third (K3) containing only three individuals (Figure S3). An additional 23 individuals showed a mixed ancestry, with membership *q*-values lower than 70% in either of these three clusters.

The geographic distribution of clusters was non-random (Figure S4). There was a significant correlation of individual assignment probability (*q*-matrix) for the K1 cluster to longitude (*r* = 0.27; *p* = 0.009) and latitude (*r* = − 0.21; *p* = 0.042). This was in contrast to the K2 cluster, where longitude was negatively correlated (*r* = − 0.24; *p* = 0.022) and correlation with latitude was not significant (*r* = 0.17; *p* = 0.11). Within the K3 cluster, no significant correlations of *q*-values to geographic variables were observed.

### Detection of outliers using LFMM

The LFMM analysis detected a total of 111 unique SNP loci showing significant association with one or more variables (Table S6). The greatest number of loci (79) was associated with phenotypic variables: spring phenology (8), height (18), and survival (53). Next, 45 SNPs appeared to be associated with geographic variables: longitude (37) and latitude (8). Finally, 36 SNP loci were associated with climate variables: PC1 (19), PC2 (12), and PC3 (5). However, there were no significant SNPs detected for altitude and autumn phenology. The highest number of common putative adaptive loci (14) was found for longitude and PC1, followed by height and survival (6).

### Detection of outlier loci based on redundancy analysis

**Fig. 3:**
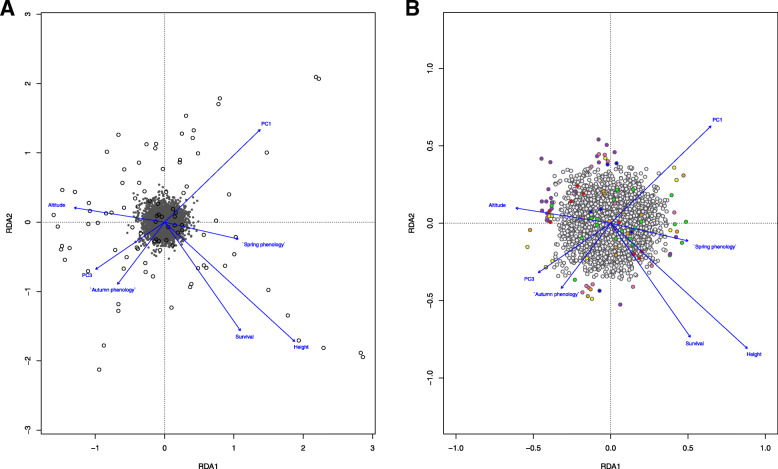
**A** Redundancy analysis (RDA) performed with 2909 SNPs (gray points in the center of plot) for 92 individuals (black circles) using geographic (altitude), climate (PC1, PC3) and phenotypic (spring phenology, autumn phenology, height, survival) variables considered as constraining variables. **B** Redundancy analysis performed using the 94 SNPs detected to be potentially associated with explanatory variables based on the RDA outlier detection approach

The RDA with 2909 SNPs was globally significant (ANOVA *F* = 1.15; *p* = 0.001) and explained about 8% of the variance (adj. *R*^*2*^ = 0.084) (Fig. 3A). This low explanatory power is not surprising because one might expect most of the SNPs in the dataset to be neutral and not to show relationships with the explanatory variables. Only the first two RDA axes were significant (RDA1, *p =* 0.007; RDA2, *p =* 0.020). Therefore, we considered the candidate loci only on the first two constrained canonical axes, which explained 18.02 and 16.57% of the genetic variation, respectively. Based on locus scores that were ± 3 SD from the mean loadings, 94 loci were identified as outliers (Fig. 3B, Table S6). The majority of candidate loci (48; 51.07%) were associated with phenotypic variables – height (21), survival (13), spring phenology (8), and autumn phenology (6). Of the remaining candidate loci, 27 (28.72%) were associated with climatic variables – PC1 (12), and PC3 (15). Finally, 19 SNPs (20.21%) were associated with a geographic variable (altitude).

Among the 111 unique SNP outliers detected with LFMM, only 4 were among the 94 RDA outliers, and 7 additional ones were located within 150 bp of one of the RDA outliers. Thus, it might be considered that about 10% of the LFMM outliers were also detected by the RDA approach. However, when considering gene models, among all 162 genes that contained significant SNPs, 27 (16.7%) had SNPs identified by both LFMM and RDA methods. Considering both analytical methods, 19, 37, and 8 loci were related to altitude, longitude, and latitude, respectively; 65, 38, 16, and 6 loci were related to survival, height, spring, and autumn phenology, respectively; and finally 30, 12, and 20 loci were related to PC1, PC2, and PC3 climatic variables (Table S6). In summary, 63 loci (51 genes) were related to geographic variables, 58 loci (49 genes) to climatic variables, and 113 loci (90 genes) to phenotypic variables (Table S6).

Interestingly, there were five genes with significant SNPs related jointly to at least one variable from each of the three categories (geography, climate, phenotype), four genes related jointly to at least one geography and one phenotype variables, and four genes related jointly to at least one climate and phenotype variables. On the other hand, there were 17 genes related jointly to at least one geography and one climate variable, which is not surprising given significant correlations among these variables (Table S4).

### Effect of geography, phenology and climate on adaptive genetic variation

A simple RDA model of combined geographic, climatic, and phenotypic variables (Model 1) explained a significant portion of variation in adaptive allele frequencies (*adj. R*^2^ = 0.15). Partial RDA was employed in order to separately determine the pure variation contributed by geographic, climatic, and phenotypic variables. Partitioning of the total variance indicated that the geographic variables accounted for 9.94% (*p* = 0.175) of the explainable total variance after removing the effect due to climatic and phenotypic variables (Model 2). The climate explained 8.70% (*p* = 0.406) of the total variance after the effect of geography and phenotypic traits was controlled (Model 3). However, the partial Model 4 showed that phenotypic variables explained 17.39% (*p* = 0.001) of the total explainable genetic variance after removing variance explained by geography and climate. In addition, 63.97% of the genetic variance could be explained by the joint effect of geography, phenotype and climate (Table 2).

**Table 2:** Partitioning of adaptive genetic variation using simple and partial redundancy analysis

Adaptive genetic variation	Interia	Percentage (%)	Pr (>F)
**Model 1: Spatial + Climate + Phenotype**	32.2	100	0.001
**Model 2: Spatial | Climate + Phenotype**	3.2	9.94	0.175
**Model 3: Climate | Spatial + Phenotype**	2.8	8.70	0.406
**Model 4: Phenotype | Spatial + Climate**	5.6	17.39	0.001
**Spatial ∩ Climate ∩ Phenotype**	20.6	63.97	NA

We also tested the relationship between individual tree heterozygosities and geographic, climatic, and phenotypic variables. It appeared that the heterozygosity determined based on all 2909 SNPs was not related to any variable. However, the individual heterozygosity based on the subset of 201 significant SNPs appeared to be significantly related to PC1 (*r* = 0.3570; *p* = 0.0005), spring phenology (*r* = 0.3047; *p* = 0.0037), longitude (*r* = 0.2177; *p* = 0.0371), and altitude (*r* = 0.2933; *p* = 0.0045). Because some of the variables are correlated, we performed a multivariate regression analysis and found that the most significant model (*adj. R*^2^ = 0.2217; *p* < 0.0001) built only with significant variables included the three geographic variables (longitude, latitude, altitude) and spring phenology. All regression coefficients were positive, indicating that putative adaptive SNPs’ heterozygosity had a tendency to increase with longitude, latitude, altitude, and spring phenology scores (the higher score, the earlier phenology), and thus towards extreme climates and earlier bud flushing.

### Additive polygenic scores

The relationships assessed between additive polygenic scores and the corresponding geographic, climatic, and phenotypic variables for each individual based on all 201 putative adaptive SNP loci are shown in Table 3 and Figures S4, S5. For almost all variables, the linear model had a lower AIC score and explained a similar proportion of variation as compared to a quadratic model (Table 3). Additive polygenic scores increased significantly with increasing latitude (*p* = 0.05), PC1 (*p* = 0.04), spring phenology (*p* = 0.01), and height (*p* = 0.01), but they decreased with decreasing longitude (*p* = 0.05) and PC3 (*p* = 0.03) (Figures S4, S5). The analyses revealed non-significant correlation of polygenic scores with altitude, PC2, autumn phenology, and survival.

**Table 3:** Correlation coefficients and *p*-values of the relationship between the additive polygenic scores and geographic, climate and phenotypic variables. The correlation coefficient and *p*-values were calculated following a linear and a quadratic model

	Linear model			Quadratic model		
Variable	*R*	p-value	AIC	*R*^2^	p-value	AIC
**Longitude**	- 0.20	0.05	625.36	0.04	0.12	728.72
**Latitude**	0.20	0.05	454.86	0.04	0.16	729.34
**Altitude**	- 0.08	0.41	1341.86	0.01	0.65	732.24
**PC1**	0.21	0.04	477.52	0.09	0.01	723.73
**PC2**	- 0.09	0.39	430.60	0.01	0.66	732.26
**PC3**	- 0.22	0.03	365.22	0.05	0.08	727.88
**Spring phenology**	0.25	0.01	246.49	0.07	0.04	726.52
**Autumn phenology**	0.11	0.30	263.82	0.01	0.51	731.73
**Height**	0.26	0.01	271.89	0.13	0.002	720.16
**Survival**	0.17	0.10	275.44	0.07	0.04	726.30

### Candidate SNPs under selection at a finer scale

**Fig. 4:**
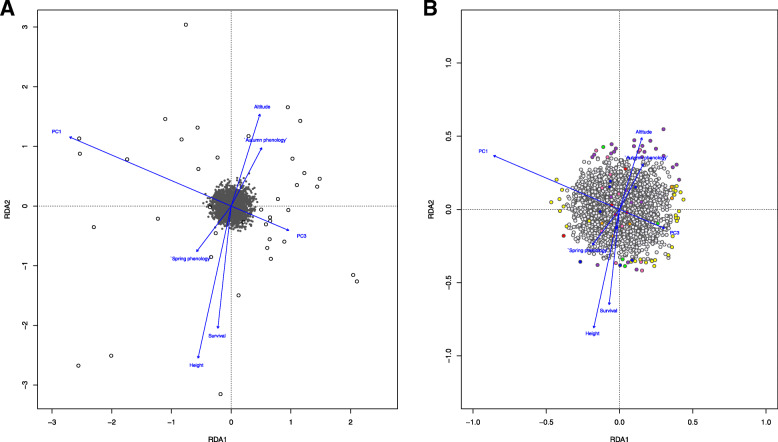
**A** Redundancy analysis (RDA) performed within the K1 genetic cluster with 2909 SNPs (gray in the center of plot) for 92 individuals (black circles) using geographic (altitude), climate (PC1, PC3) and phenotypic (spring phenology, autumn phenology, height, survival) variables as constraining variables on the first two ordination axes; **B** plot showing candidate loci detected based on locus scores ±3 SD from the mean loading on each RDA axis coloured by the most highly correlated explanatory variable

Within the K2 cluster observed predominantly in Western Europe, RDA was not significant (ANOVA *F* = 1.01; *p* = 0.27) and the proportion of variance explained by the predictor variables was 10% (adjusted R^2^ = 0.10), thus we did not investigate candidate loci within this region. On the other hand, in the K1 cluster predominant in southeastern sampled populations, the proportion of total genetic variance explained by the predictor variables was similar (*adj. R*^2^ = 0.11), however the RDA was found to be significant (ANOVA *F* = 1.05; *p* = 0.004) (Fig. 4A). The proportion of total genetic variance explained by the predictor variables was lower than that observed for the total dataset. As with the broader scale, we considered candidate loci on the first two constrained axes, which explained 16.65 and 15.03% of the genetic variance, respectively. We detected 62 candidate loci (Fig. 4B). The majority of candidate loci (30; 48.39%) were associated with climate variables (PC1 (28) and PC3 (2)). Of the remaining candidate loci, 29 (46.77%) were associated with phenotypic variables (height (21), survival (5), spring phenology (1), and autumn phenology (2)) and 3 (4.84%) were associated with altitude. Only eight SNP loci detected over the regional scale were also significant at a global scale. However, 13 SNPs were located in 10 genes also identified by the global RDA.

### Functions of genes with significant SNPs

BLAST searches of candidate genes against the GenBank non-redundant protein database (NR) and also UniProt’s Swiss-Prot and TrEMBL protein databases revealed that most of the candidate genes in the current study had putative gene product names and general descriptions. We assigned functions to the candidate genes with the Gene Ontology (GO) classification, which provides a standardized set of terms to describe the genes and gene products of different species. Details of all genes initially selected for this study along with their product names and related GO terms can be found in Table S2.

**Fig. 5:**
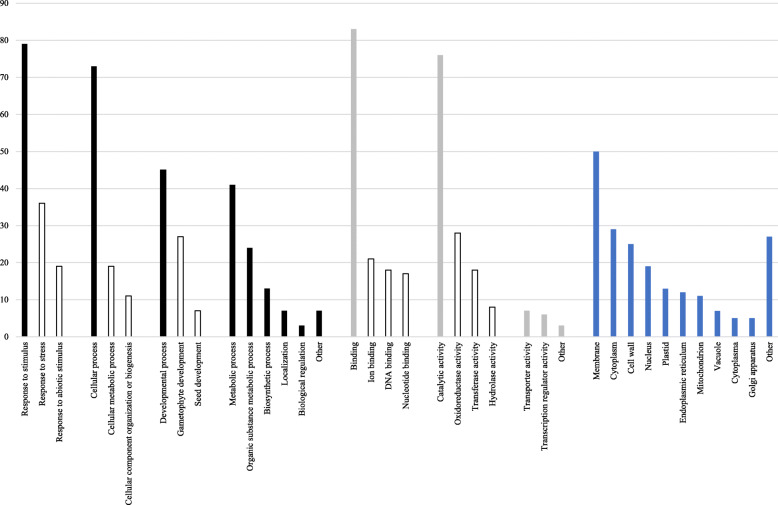
Gene ontology classification of genes with significant SNPs. The following GO slim terms contributed to the annotation of the 162 genes: biological process (black), molecular function (grey), cellular component (blue). The most abundant GO terms within the most common GO slim terms are indicated as white bars

All significant SNPs were related back to the gene through which they were found to assert their putative adaptive function. In total, the 255 unique significantly associated SNPs were distributed across 162 genes. Gene ontology annotation assigned at least one GO term for 116 (71.6%) of these genes. The total number of GO terms was 615. Among them, 86 genes were associated with 255 ‘biological process’ GO terms, 88 with 175 ‘molecular function’ GO terms, and 96 with 185 ‘cell component’ GO terms (Fig. 5). The most abundant GO slim terms were ‘response to stimulus’, ‘binding’, and ‘membrane’ in terms of biological process, molecular function, and cellular component, respectively.

## Discussion

Understanding the genomic background of adaptive traits is particularly important for forest tree species occupying wide and climatically variable geographic areas [93], such as *Fagus sylvatica*. Until now, there have been only a few attempts to investigate signatures of adaptation based on SNP polymorphism in candidate genes in beech. These attempts usually included a limited number of candidate genes and were focused on populations originating from relatively restricted areas [69, 91]. In this study, we applied a mixed approach where first we selected candidate genes differentially expressed during dormancy release, as identified by Lesur et al. [89], and supplemented these with a list of additional candidate genes known to be related to bud burst in plants. Then, we designed the sequence capture experiment to study the SNP polymorphism of these genes across a wide range of *F. sylvatica* distribution and their associations to geographic, climatic, and phenotypic variables.

### Methodological constraints

There are two main weaknesses in our study: a relatively small sample size (mostly one sample per population), and the use of population-wide phenotypic measures instead of exact phenotypes of individual sequenced trees (which prevented us from performing typical GWAS analyses). These weaknesses could have restricted our ability to find significant genomic associations. However, because we used mostly one sample per population, such averages of population characteristics are better representations of populations than individual measures. Note also that survival can be assessed only at a population level. Nevertheless, despite these limitations, in combining the same univariate and multivariate approaches to find GEA and GPA, we identified 255 unique loci related to 162 genes exhibiting adaptive significance. These loci came from a dataset of 2909 SNPs derived from targeted sequencing of 380 phenology-related genes. These SNPs exhibit associations with environmental variables hypothesized to influence spatially varying selection in European beech, or associations with phenotypic traits (phenology, height, survival) related to adaptation. Moreover, different genotype-environment associations were identified within the Southeastern Europe region as compared to the entire sampled range of European beech, implying that selection pressures may differ across spatial scales. Our results demonstrate that, in beech, environmental conditions play important roles as drivers of genetic diversity of genes related to adaptive potential, thereby facilitating local adaptation of the species.

### Genetic diversity and population structure

Genetic diversity in relevant genes is the basis for adaptation to environmental stress [94, 95]. In this study, we found that genetic diversity based on 2909 SNP markers was relatively high (He = 0.290) and comparable to other studies on beech [65, 91, 96, 97]. The high genetic diversity revealed for European beech might be a good basis for the capacity of adaptation to environmental stress. We observed that the individual heterozygosity estimated on the basis of significant SNPs increased with latitude, longitude, and altitude (i.e. towards extreme climates, where a future beech expansion is expected [98]) and earlier phenology, indicating the importance of relevant genes in local adaptation.

The STRUCTURE analysis revealed a weak population structure of beech in Europe. These findings are in line with recent studies at a regional scale reporting low population structure for beech populations in France [63], Germany [91, 97, 99] and Switzerland [100]. The low genetic structure of European beech in Central Europe may result from specific postglacial recolonization patterns from one predominant refugial area, and from high gene flow among populations [101, 102].

### Detection of loci affected by selection

Many important phenotypic traits (e.g. flowering time, dormancy, chilling requirement, seed stratification) are under polygenic control, where several loci exert small effects [80, 103, 104]. Lesur et al. [89] identified in beech a number of genes differentially expressed during dormancy release, but, until now, it was unclear whether these genes were variable, and whether their variation was important for local adaptation.

Here, we found that 162 of these genes (among 380 tested) appeared to be significantly related to at least one of the explanatory variables related to geography, climate, or adaptive phenotypic traits. An attempt to relate the phenology-related genes to geographic, climatic, and a subset of adaptive traits seems reasonable because, in beech, phenology (in a broad sense) varies widely along geographic and climate clines [105].

Among climate variables, the largest number of outlier loci (30 SNPs) was related to the first principal component (PC1). Our results suggest that the first principal component of the PCA is an important predictor of genetic variation at candidate loci, and thus may be a potential driver of spatially variable selection for European beech. Indeed, this PC accounted for 43.05% of the variance among the 19 bioclimatic variables. Analysis of correlations between PC1 and bioclimatic variables (Table S3) suggests that the genes containing SNPs exhibiting strong associations with PC1 may therefore be under selection from temperature and water availability limitations. However, since PC1 was significantly correlated with several bioclimatic variables, it is difficult to ascertain exclusively the exact agent of selection responsible for the patterns observed in this study.

The timing of bud burst in populations of temperate trees is determined mainly by temperature and genetics [106–110]. It is possible that temperature may not be the only direct causative agent of selection, but instead, adaptation may be directly attributable to other variables that are correlated with temperature, such as water availability.

Another strategy to find genomic signatures of selection is to test for associations between genotypes and traits based on potentially differentiated populations growing in a common environment, as in common garden tests [11]. Such genotype–phenotype association analyses have the advantage of establishing more direct links between genes and traits that are potentially under selection [111]. In this study, in total 113 unique outliers were significantly associated with adaptive traits of European beech (autumn phenology, spring phenology, height, and survival), which explained 17.4% of the total outlier genetic variance after accounting for spatial and climate effects in a conditioned RDA. However, 16 SNPs were related directly to spring phenology. This number is higher than in the results of Müller and coauthors [91, 97], who reported only a few SNPs with significant association with bud burst in Germany. However, this may suggest significant differences in bud burst timing for the different populations in the whole natural range of European beech. This finding is in accordance with several other studies that revealed different bud burst timings for different beech populations/provenances [58, 60].

### Additive effect of outliers on environmental and phenotypic correlations

To determine the extent to which individual outlier loci collectively mirror environmental variation across the European beech distribution range, we used a multilocus approach based on additive polygenic scores for phenotypic, geographic, and climate variables [112]. The strong positive correlation observed between individual polygenic scores calculated across all significant candidate loci and the first principal component is consistent with spatially varying selection across temperature and precipitation gradients, where different alleles are maintained in different environments. However, the quadratic model indicated optimum values of PC1 ≈ 2 maximizing additive polygenic scores (Figure S6), implying that the climatically suitable habitat for European beech is the area of Europe with higher temperature and precipitation (mostly across the area of Germany, Figure S1).

Genotypic variation at outlier loci detected in candidate genes should be compared with phenotypic data to establish associations between genotype, phenotype and fitness. The correlation between individual polygenic score and bud burst phenology appeared to be significant for both linear and quadratic models (Table 2). However, the phenotypic variation explained by significant SNPs associated with bud burst was low (linear model *R*^2^ = 6%; quadratic model *R*^2^ = 7%) but comparable to the results of other studies analyzing different traits and tree species [84, 113, 114]. Noticeably, additive polygenic scores generally increased with phenology scores, favoring individuals with earlier bud burst (Figure S5, S6).

The bud burst timing in deciduous trees has important fitness implications [23]. In this study, a positive correlation between individual polygenic score and bud burst phenology suggests that selection favors genotypes with earlier bud burst. Our results indicate that the warming climate in Europe might advantage northern-origin genotypes, which is further supported by a positive correlation between individual polygenic scores and latitude. It well corresponds with the predictions of Saltre et al. [98], i.e. that the climatically suitable habitat for European beech will shift north-eastward because the expected increase of temperature and precipitation at the range’s northern margins will increase survival and fruit maturation success.

### Differentiation processes in European beech at regional scale

Within the K1 genetic cluster, predominant in southeastern populations, PC1 was the most strongly associated predictor of genetic variation at candidate loci, given both the number of candidate loci associated with climate predictors (PC1, PC2, PC3) and the strength of correlations. On the other hand, 45.8% of outliers at the regional scale were related to phenotypic variables. However, there was a lack of correspondence between the list of outlier SNPs at the regional and the broader scale. If taken as true positives, the genotype-environmental associations presented here likely represent loci affecting phenotypic traits to different degrees in different climatic conditions.

European beech can be considered as a late-flushing species growing under warm or mild climates, but not necessarily when exposed to colder climates within its range [59, 115]. This can be attributed to the known effects of winter chilling temperatures on the ability to bud burst in spring, where an increased duration of chilling temperatures reduces the thermal requirement for bud break [116–119]. As our beech samples related to K1 (predominantly southeastern populations) came from a different climate, we could then hypothesize that the lower number of genes involved in phenology compared to Europe as a whole may be explained by the delayed bud burst observed in beech [120]. However, a limited number of studies exist on the possible effect of water availability on bud burst timing of temperate forest trees [121, 122]. Both factors could potentially lead to adaptive divergence in phenology to cope with global warming conditions, and should be investigated further. The strong effect of PC1 (in terms of both the number of SNPs as well as the significance of correlations with additive polygenic scores) indicates that an environmental gradient affecting the selection of phenology-related genes lies across the western-eastern direction.

### Physiological importance of the genes under selection

A number of candidate genes identified as significant in this study were already investigated in earlier works on European beech. These include: aldehyde dehydrogenase [62]; alcohol dehydrogenase [63]; protein phosphatase 2c [63, 65, 68]; chlorophyll a/b-binding protein [63, 70]; heat shock protein [63, 68, 69]; short-chain dehydrogenase reductase; 3-ketoacyl-CoA synthase 11; and dehydration-responsive element-binding protein 1b [68].

A detailed discussion of all genes found to be significantly associated to climate, geography, or phenotype variables is beyond the scope of this paper. However, we focused on a subset of genes significantly related to spring phenology. The monitoring of bud burst extended from the dormant bud stage (1) to the stage of leaves being stretched, smooth, and shiny (7). Therefore, the ‘bud-burst score’, as presented in this study, should be considered a complex phenotypic trait. Potential roles of specific genes controlling stages 1 and 2 (i.e. the dormant and the swelling bud) are related to release from ecodormancy. Conversely, genes preferentially expressed at the end of bud burst should be considered as candidate genes for the stages of leaf development and tissue elongation rather than for bud burst.

Cell defense and rescue-associated genes are expressed during the dormancy stage and at the onset of bud burst [36, 123–126]. This is confirmed in our study by functional analysis of genes with significant SNPs, where the most abundant GO slim term was ‘response to stimulus’, in terms of biological process (Fig. 5). Many of these genes encode proteins associated with detoxification, cold/drought hardiness, and pathogenesis, and were reported to be induced during dormancy in *Quercus petraea* [36] and *Populus deltoides* [127]. Growth inhibition occurs at the dormancy stage [128], as exemplified by the association between a gene encoding the protein *TIFY9* and spring phenology, and also altitude. After dormancy release, buds remain cold-acclimated until a period of warm temperature results in deacclimation and bud break [129]. Therefore, pathogen-resistant proteins such us NDR1, GLOX, and phosphoribulokinase probably have antifreeze activity [130]. In addition, we found significant associations with genes encoding dehydrin (Dehydrin COR47), heat-shock proteins (22.0 kDa class IV heat shock protein, 18.1 kDa class I heat shock protein, Stromal 70 kDa heat shock-related protein, chloroplastic), heat stress transcription factor C-1 (HSFC1), and also 10 kDa chaperonin (mitochondrial) which are all related to phenological protection against desiccation and temperature stresses through the accumulation of dehydrins and heat-shock proteins at the onset of flushing.

We found a significant association between UDP-glycosyltransferase 83A1 and phenotypic and climatic variables. The UDP-G gene plays a role in carbohydrate metabolism, and was already reported to be related to bud burst, drought, and soil clay content [64, 89, 91]. It is also well-known that water stress enhances the production of reactive oxygen species in plants and increases susceptibility to pathogens [131]. On the one hand, oxidative stress, counteracted by the accumulation of dehydrins, heat shock proteins or sugars could contribute to the protection of cells against oxidation [132]. On the other hand, NDR, GLOX, and phosphoribulokinase genes may also contribute to defense against pathogens.

Genes associated with the gene ontology (GO) terms ‘regulation of growth’ and ‘regulation of unidimensional cell growth’ such as HD16 (Casein kinase 1-like protein HD16) and CLASP (CLIP-associated protein), respectively, may be related to the resumption of mitotic activity in meristematic cells. Indeed, it is well-known that bud burst is essentially driven by a resumption of mitotic activity in meristematic cells [133]. However, functions of these genes during bud burst are unclear. Casein kinases are involved in regulating flowering time through gibberellin signaling and are required for normal development of male floral organs and grains via the modulation of gibberellin signaling [134]. Nevertheless, CLIP-associated protein is required for cell morphogenesis and cell division [135, 136].

Activity of glycosyl hydrolases, such as probable glucan endo-1,3-beta-glucosidase A6, were also significantly associated with bud burst of European beech. This particular +enzyme is induced by gibberellic acid and is known to play a role in cell-wall mobilization and cell elongation [137] through hydrolysis of glycosidic bonds linking cell-wall components. During bud burst, induction of this enzyme could thus reflect the initiation of outgrowth taking place in early stages of bud burst. Another regulator of carbohydrate modification, namely a 1,4-alpha-glucan-branching enzyme, was also associated with bud burst phenology. Starch branching enzymes catalyze the formation of α-1,6 branches within α-glucan chains by cleaving internal α-1,4 links followed by re-attachment of the cleaved glucan to another chain via an α-1,6 linkage [138]. This enzyme was highly expressed in oak at the dormancy stage [139], suggesting that the hydrolysis of storage starch or glycogen is repressed in the quiescent buds. In contrast, the reduction in this gene expression upon bud swelling would indicate the onset of starch mobilization at this developmental stage.

In general, the contribution of genes essential for energy supply can be observed at the end of bud burst, as shown by genes encoding cytochrome P450 704B1, cytochrome b561 DOMON domain-containing protein At5g47530, and also CAB6A (chlorophyll a/b-binding protein 6A). In parallel, ribulose bisphosphate carboxylase/oxygenase activase (responsible for the activation of RuBisCO and RuBisCO large subunit-binding protein subunit beta, chloroplastic), was also found in this study, as well as that of two genes with the molecular function of glyceraldehyde-3-phosphate dehydrogenase (NAD+) (aldehyde dehydrogenase family 7 member A1 and aldehyde dehydrogenase family 2 member B4), as previously reported by Wang et al. [140]. Indeed, these authors demonstrated that enzyme activity of the glycolytic pathway increased at the release of dormancy. Regarding the developmental stages of spring phenology used in this study, this contribution of energy-related genes is undoubtedly caused by leaf development, which starts at stage 4, but mainly develops at stages from 5 to 7.

## Conclusions

We investigated whether genes involved in the release of dormancy in *Fagus sylvatica*, a widely distributed forest tree species growing in various climates, show evidence of selection signatures in relation to geographic, climatic, or phenotypic variables related to adaptation. Using 380 candidate genes and a sequence capture approach, we identified 2909 associated SNPs and, based on two complementary analytical methods (LFMM, RDA), we narrowed this down to 201 SNPs within 162 (42.6%) genes associated with geographic, climatic, or population-wide phenotypic variables related to adaptation. This significantly extended the existing list of genes putatively identified as adaptive and related to spring phenology. A large proportion of significant genes seems not surprising given that the timing of spring phenology (or dormancy release, in general) varies along geographic and climate clines and has a strong adaptive importance. However, the variable pattern of significant loci across different spatial scales suggests that local adaptation may occur through multiple mechanisms, and that the importance of different genes may depend on the spatial and environmental context.

## Material and methods

### Plant material

We sampled one to four individuals (92 in total) of European beech originating from 47 provenances (geographic and climate data points) growing in the common-garden experimental site located in Siemianice Experimental Forest District in south-central Poland (Fig. 1; Table S1) [141]. Freshly developed leaves were sampled from individual plants in early spring 2015. After collection, leaf samples were labeled, dried, and kept at room temperature until DNA extraction.

### Environmental data

Environmental data for the locations of the 47 provenances was downloaded from the WorldClim database [142]; http://www.worldclim.org/) from maps with a spatial resolution of 30 arc-seconds (approximately 1 km^2^) using DIVA GIS 7.5 software [143]. The characteristics of each location were quantified using a set of nineteen bioclimatic variables representing annual trends (e.g. mean annual temperature, annual precipitation), aspects of seasonality (e.g. annual range in temperature and precipitation), and extreme or potentially limiting environmental factors (e.g. temperature of the coldest and warmest months, and precipitation of the wettest and driest months). Since several bioclimatic variables are collinear in nature, we applied principal component analysis (PCA) to extract essential bioclimatic information with focused indices using the R package FactoMineR [144]. PCA is a useful approach in reducing the number of dimensions of explanatory variables with acceptable information loss under most conditions [145], and it has been used in other landscape genomics studies [29].

### Phenotypic variables

The indices of spring and autumn phenology, tree height at 5 years of age, and survival in each provenance were obtained from Barzdajn and Rzeźnik [141]. These represent average and standardized observations. Spring phenology was assessed between 1995 and 1998 (on a seven point scale, assessed on a single day in each year), while autumn phenology, tree height, and survival were measured in 1998 [141]. Each individual sampled in this study was assigned a respective phenotypic index determined for its provenance (Table S1). To identify relationships between phenotypic, geographic, and climate variables, pairwise Pearson’s correlation and canonical correlation analyses (for the groups of variables) were performed, using STATISTICA software.

### DNA isolation, candidate genes and genotyping

Total DNA was extracted from sampled individuals using the GeneMATRIX Plant & Fungi DNA Purification Kit (EURx, Poland) according to the manufacturer’s protocol with minor modifications. Extracted DNA was quantified using an Eppendorf BioPhotometer and Quantus Fluorometer (Promega). 100 ng of DNA from each tree was used for individual exome capture and sequencing.

We investigated the genetic variation that might be associated with geographic, climate, or phenotypic variables, using an exome capture approach [146] based on an initial set of 485 candidate genes (Supplementary Table S2). The candidate genes were selected as follows. Firstly, we created a set of 421 genes from the previously published *F. sylvatica* transcriptome (FSV2, [89]). These 421 genes were differentially expressed during bud dormancy release, as identified by the EdgeR method described by Lesur et al. [89]. Secondly, we chose 64 additional candidates based on their appearances in other published studies which aimed to identify genes of potential adaptive importance related to drought stress (53) and photoperiod (11) in beech and other species. These selected sequences from *F. sylvatica* [147], *Populus trichocarpa* [42, 148], and *Pieca abies* [149] were verified by BLAST software (Washington University Basic Local Alignment Search Tool Version 2.0) to find corresponding homologous sequences in the beech transcriptome (FSV2, [89]). Next, we ran BLAST searches to annotate the candidate genes. We aligned our sequences using BLASTX with an e-value cut-off of 0.0001 against GenBank’s non-redundant protein database (NR), UniProt Swiss-Prot, and TrEMBL protein databases. Information about these matching sequences was used to annotate our candidate genes. For significant matches in the databases, we extracted gene names, general descriptions, Gene Ontology (GO) categories, and additional information from the UniProt knowledge database.

DNA library preparation and exome capture was performed using the SeqCap EZ Library SR User’s Guide v5.1 (Nimblegen Roche). However, because the reference genome of *F. sylvatica* was not available at the time of the experiment set-up, the bait development performed in SeqCap EZ library preparation utilized only the published cDNA sequences for the 485 genes [89]. Following capture, 125 bp paired-end sequencing of 92 samples was performed on a single lane of Illumina HiSeq2500 sequencer. Sequence capture and sequencing was performed by IGA Technology Services (www.igatechnology.com; Udine, Italy).

Sequence reads were assessed with FastQC software [150]. Adapter sequences were removed with cutadapt [151], and contaminant sequences (e.g. chloroplast genome) were removed with ERNE-FILTER [152]. Filtered, high-quality reads were mapped to the European beech reference genome version 1.3 [90] using Burrows–Wheeler Aligner (BWA-MEM algorithm) with default settings [153]. SAM files were converted to BAM files, sorted, and indexed using SAMtools v.0.1.19 [154]. SNPs were called with the Heap v.0.7.8 software [155] and stored in the *vcf* format.

Filtering of variants was carried out using VCFtools [156] with the following cut-offs: minimum depth of 10 reads; minor allele frequency > 5%; missing data per SNP < 25%. Contigs containing fewer than 10 base pairs per SNP were removed to control for mapping errors. Linkage disequilibrium (LD) among SNPs was accounted for using the LD pruning tool in Plink 2.0 [157, 158], where independent pairwise comparisons between each SNP were made and an r^2^ value was calculated. A cut-off of r^2^ > 0.5 was used, whereby one of a pair of SNPs was removed from the dataset if the coefficient of determination between the pair was > 0.5, thus removing SNPs showing strong signals of LD.

SnpEff software [159] was employed to classify SNPs according to their genomic regions and their positions relative to capture target regions. We identified the coordinates of targeted regions in the reference genome of European beech [90] based on gene models. Gene models were created by mapping and aligning candidate gene sequences to the genome using BLAT [160] and GMAP software [161] with 95% identity and 90% coverage cut-offs.

To improve gene annotations, we performed a manual curation of gene models. While sequence capture technology primarily targets exons, many functional elements are located outside the exonic regions. It is well-known that SNPs located in promoter and UTR regions may regulate gene expression. It is traditionally considered that introns are not as important as exons, but several studies have demonstrated some functional significance for introns [162, 163]. Many genome-wide association analyses (GWAS) and genotype-environment association analyses (GEA) have suggested strong associations between intergenic SNPs and phenotypic or environmental variables [43, 164]. Therefore, because these data should not be ignored in GEA studies [165], we analyzed SNPs located in exons, introns, and sequences up to 500 bp away from each candidate gene. Finally, the SNP dataset was filtered to remove remaining non-target variants using BEDtools v. 2.28.0 [166].

### Copy number assessment

Copy number variations (CNVs) were identified using CNVkit [167], a piece of software designed to assess log_2_ copy ratios from targeted capture NGS data based on reads mapped to both on-target and off-target regions. CNVkit has the highest sensitivity and a typical specificity for small CNVs with sizes below 100 kb [168]. Therefore, CNVkit seemed to be the best suited to our data. CNVkit was run with the default parameters of the *batch* command after creating a flat reference genome as suggested in the manual using the command *reference*. A threshold of 0.2 was applied to identify the signals for amplification and deletions of the genes.

### Genetic diversity and structure

Genetic diversity was estimated based on expected heterozygosity (He) defined as the probability that two randomly chosen alleles from the population are different using PowerMarker 3.25 software [169]. We investigated the genetic structure of sampled individuals by applying the Bayesian model-based clustering method implemented in the software STRUCTURE 2.3.4 [170]. The number of assumed populations (*K*) was set from 1 to 10, carried out each time in sets of 10 repeats, with a burn-in period of 50,000 iterations and 100,000 MCMC (Markov Chain Monte Carlo) repeats, and an admixture model with correlated frequencies. The optimal value of *K* was determined based on the *ΔK* method [171] using the software STRUCTURE HARVESTER 0.6.94 [172]. The probabilities of individuals’ assignments to particular clusters (*q*-matrix) were used to test the correlations with geographic variables of the origin of populations sampled.

### Detection of outliers using LFMM

Because phenotypic variables were represented by population averages instead of individual measures, we applied the same approaches (LFMM and RDA) to detect loci with significant effects related to geographic, climate, and phenotypic variables. We used the latent factor mixed model (LFMM) approach [173] to find candidate loci under selection. According to P de Villemereuil, É Frichot, É Bazin, O François and OE Gaggiotti [76], LFMM is expected to provide the best compromise between power and error rate across different analytical scenarios. LFMM is also known to be less susceptible to both false negatives and false positives [173, 174] than other genotype-environment association (GEA) methods, such as Bayenv2 [175], because it does not rely on a specific demographic model when accounting for population structure [76, 174].

We employed an MCMC algorithm for regression analysis whereby potentially confounding population structure is modeled with unobserved (latent) factors [176]. As missing data can reduce the power of association studies [177, 178], we imputed the missing data based on the ancestry coefficients estimated by sNMF, using the “impute” function from the R package LEA [176]. In sNMF, we set *K* based on the number of distinct genetic clusters identified following the population genetic structure analysis and kept the best out of 10 runs based on a cross-entropy criterion. The MCMC algorithm was used for each of the geographic, climate, and phenotypic variables (i.e. longitude, latitude, altitude, PC1-PC3, spring and autumn phenology and height), using 50,000 steps for burn-in and 100,000 additional steps to compute LFMM parameters (*z*-scores) for all loci. The number of latent factors was set at the identified value of *K*. In order to compensate for run-to-run variation, the analysis was repeated over 10 independent runs and *z*-scores across runs were then combined in R using the LEA package [176]. The LEA package was also used to adjust *p*-values for multiple testing using the Benjamini–Hochberg procedure, and to calculate the genomic inflation factor to modify *z*-scores allowing for the control of the FDR, as described in E Frichot and O François [176]. A list of candidate loci with an FDR of 1% and adjusted *p*-values of < 0.001 was then generated for each explanatory variable.

### Redundancy analysis to detect outlier loci

To test the multivariate relationships between genetic variation with geographic, climate and phenotypic variables, we conducted redundancy analysis (RDA) using the vegan 2.4–5 package in R [179]. RDA is a two-step process in which predictor (geographic, climatic, or phenotypic) variables and the response (the matrix of allele frequencies) variables are analyzed using multivariate linear regression, producing a matrix of fitted values. Next, PCA of the fitted values is used to produce constrained axes, which are linear combinations of the predictors [180].

RDA was used to estimate the proportion of variance in allele frequencies at SNPs across all sampling locations, which could be explained by geographic, climate, and phenotypic predictors based on the adjusted r^2^. We removed longitude, latitude, and PC2 variables from analyses because they were highly correlated (|r| > 0.7) [181] with PC1 (for longitude) and altitude (for latitude and PC2). Since the RDA method requires complete datasets, we performed an imputation of missing genotypes using the most common genotype at each SNP across all individuals. The global RDA’s significance was tested with an analysis of variance (ANOVA) following 1000 permutations. The explicative importance of the variables was represented as vectors in biplot graphs. Next, the candidate loci were identified based on locus scores (i.e. the loading of each locus in ordination space) separated by ±3 SD from the mean loading on the first two constrained ordination axes [77]. Predictors exhibiting the strongest associations with each candidate adaptive locus were identified using Pearson’s correlation coefficients (r). We performed a second RDA analysis using the same methods as described above, but this time within genetic groups identified by STRUCTURE independently in order to determine whether selection pressures vary between the genetic clusters.

### Additive polygenic scores

The additive polygenic scores approach was used to assess the cumulative signal of candidate loci in response to environmental variation [112]. Firstly, alleles across all candidate loci that were associated with increasing values of a given geographic, climatic, or phenotypic variable (i.e. longitude, latitude, altitude, PCs, spring and autumn phenology, and also height) were identified based on the sign of their correlation between allele frequencies and the variables. Polygenic scores for each individual tree were obtained by summing the number of favored alleles for a given trait over loci. The correlation between individual additive polygenic scores and each geographic, climatic, or phenotypic variable was tested to evaluate how the cumulative signal of candidate adaptive alleles varied with each explanatory variable. Two models (a linear and a quadratic) were tested for each variable, and the best-fit model was determined based on the lowest Akaike information criterion (AIC) value.

### Effect of geography, climate and phenotype on adaptive genetic variation

Partial redundancy analyses (pRDA) were performed on a combined dataset comprising outliers detected by both the RDA and the LFMM approaches to examine the relative contribution of geographic, climatic, and phenotypic variables, along with their combinations to adaptive genetic variation. Four different models were considered for partitioning variance components of the RDA (Table 3): Model 1 – a simple model with all geographic, climatic, and phenotypic variables given as explanatory variables; Model 2 – a partial model in which genetic data is explained by geographic data conditioning on climatic and phenotypic variables; Model 3 – a partial model where the genetic data is explained by climate conditioned on geographic and phenotypic variables; and Model 4 – a partial model in which the amount of genetic variation is explained by phenotypic variables conditioning on geographic and climatic data. Partial RDA was carried out using the vegan 2.4–5 package in R [179] with significance determined based on 999 permutations.

## Supplementary Information

**Additional file 1: Table S1.** Description of sampled populations. **Table S2.** List of 485 candidate genes and their functional annotations. **Table S3.** Correlations between 19 environmental variables (BIO1-BIO19) and the first three principal components. **Table S4.** Pearson’s correlations (below diagonal) between geographic, climate and phenotypic variables, and canocical correlation coefficients (above diagonal) among groups of variables. Coefficients significant at *p* < 0.05 are indicated by bold-italic face. **Table S5.** List of valid gene models and their coordinates in the reference genome of European beech (Mishra et al. 2018). **Table S6.** List of outlier loci and their functional annotation across the entire sampled geographic area and within regional groups.

**Additional file 2: Figure S1.** Distribution of coefficients of three first principal components (PC1, PC2, PC3) determined based on 19 bioclimatic variables. **Figure S2.** Graphical method (as in Evanno et al. 2005) allowing for detection of the number of groups K using (A) ΔK and (B) the rate of change of the likelihood distribution (mean log-likelihood values). **Figure S3.** Bar plot of admixture proportions of individuals, inferred using K = 3 based on 2909 SNP loci. Individual’s proportions (*q*-values) are sorted within each cluster (cluster K1 – red, K2 – blue, K3 - green. **Figure S4.** Geographical distribution of European beech clusters according to the K = 3 model in STRUCTURE. **Figure S5.** Relationships between additive individual polygenic scores based on all 201 outlier markers and each of the explanatory variables: geographic (longitude, latitude, altitude), climate (PC1, PC2, PC3) and phenotypic (spring phenology, autumn phenology, height, survival). The solid line represents the regression line of the linear model. **Figure S6.** Relationships between additive individual polygenic scores based on all 201 outlier markers and each of the explanatory variables: geographic (longitude, latitude, altitude), climate (PC1, PC2, PC3) and phenotypic (spring phenology, autumn phenology, height, survival). The solid line represents the regression line of the quadratic model.

## Data Availability

Illumina data are publicly available at the NCBI BioProject PRJNA721723 (https://www.ncbi.nlm.nih.gov/bioproject/721723). Filtered SNP data in VCF format for each geographic context are available from the corresponding author upon request.
