# Supplementary material for: Genomic signatures of natural selection at phenology-related genes in a widely distributed tree species Fagus sylvatica L
Source: BMC Genomics. 2021 Jul 31;22:583. doi: 10.1186/s12864-021-07907-5 (PMC8325806; doi:10.1186/s12864-021-07907-5)
Supplement: Supplementary file 1 — Additional file 1: Table S1. Description of sampled populations. Table S2. List of 485 candidate genes and their functional annotations. Table S3. Correlations between 19 environmental variables (BIO1-BIO19) and the first three principal components. Table S4. Pearson’s correlations (below diagonal) between geographic, climate and phenotypic variables, and canocical correlation coefficients (above diagonal) among groups of variables. Coefficients significant at p < 0.05 are indicated by bold-italic face. Table S5. List of valid gene models and their coordinates in the reference genome of European beech (Mishra et al. 2018). Table S6. List of outlier loci and their functional annotation across the entire sampled geographic area and within regional groups. [file 12864_2021_7907_MOESM2_ESM.docx]

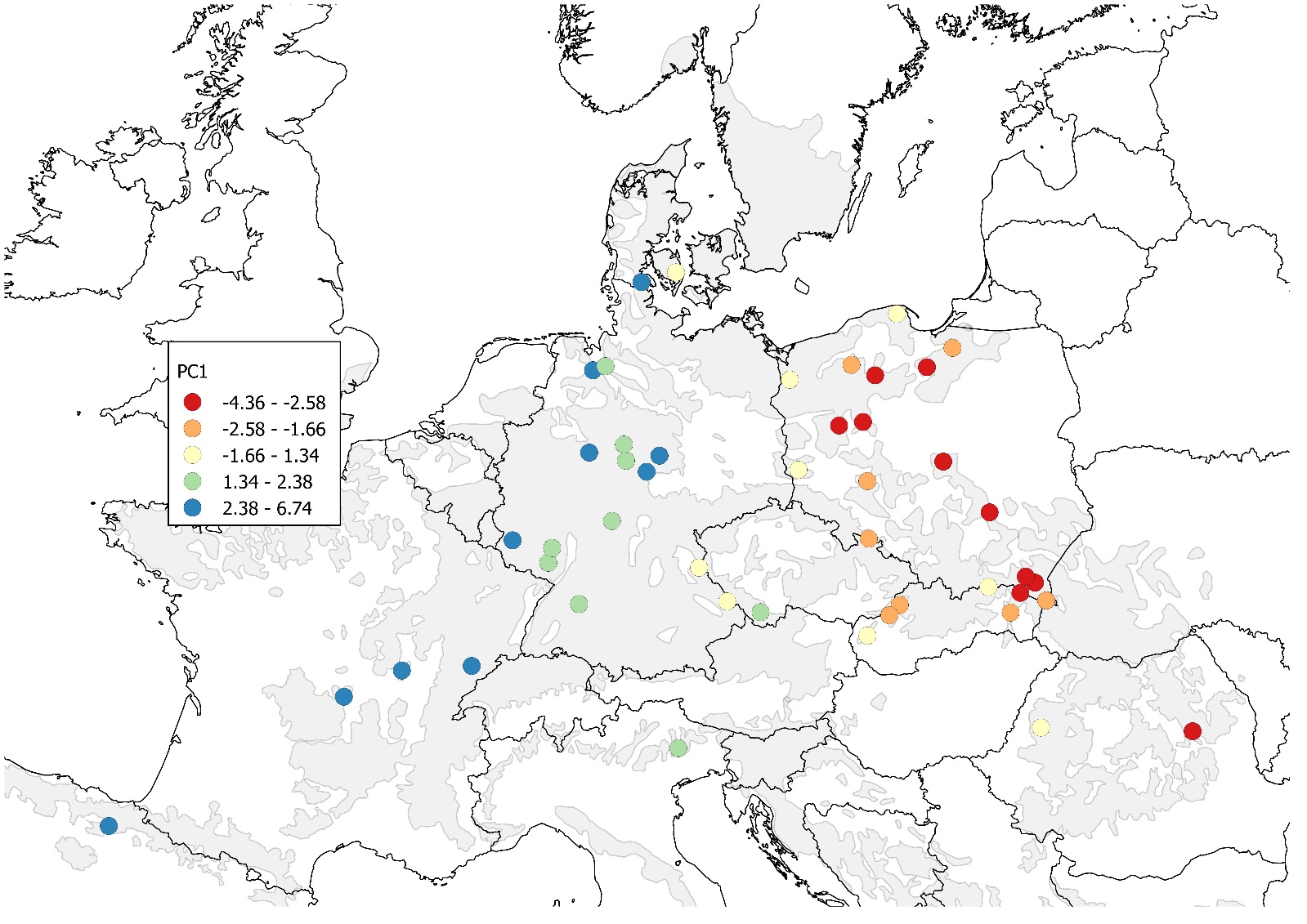

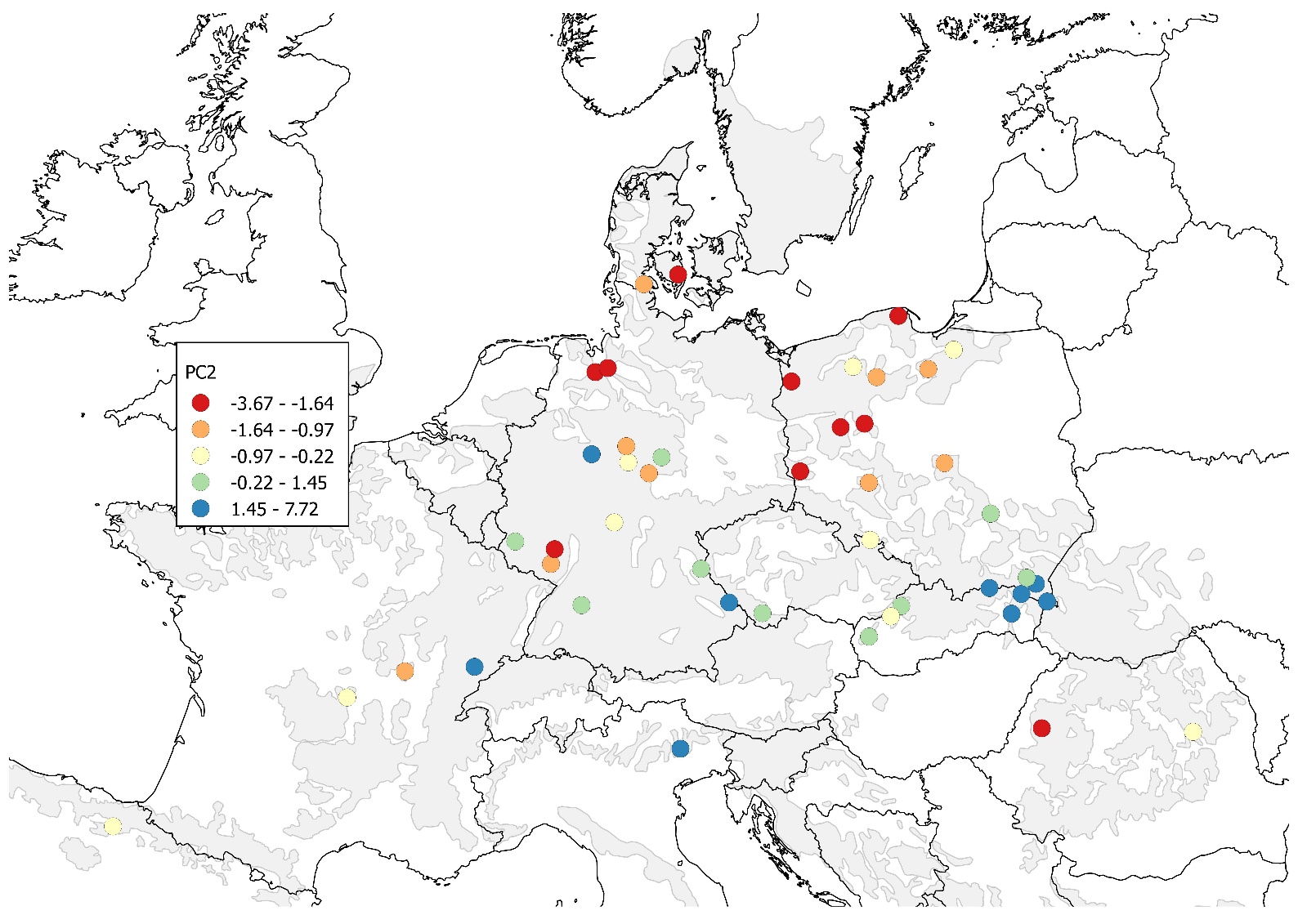

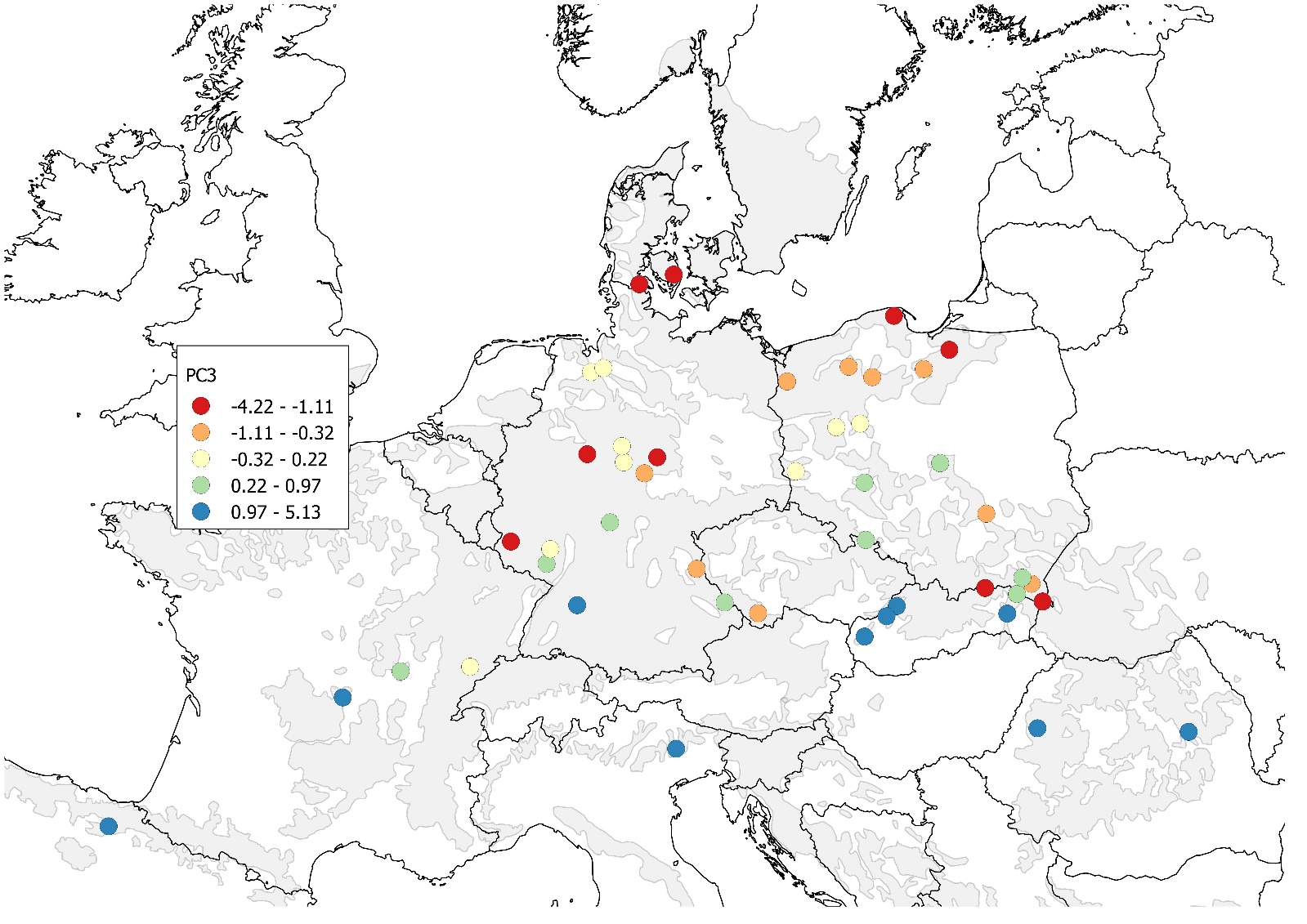


**Figure S1.** Distribution of coefficients of three first principal components (PC1, PC2, PC3) determined based on 19 bioclimatic variables.

| **A**  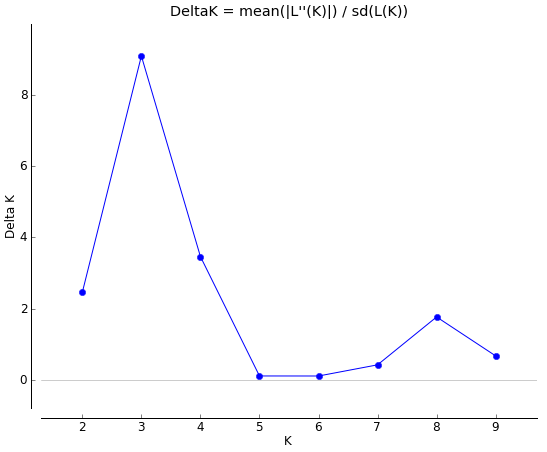 | **B**  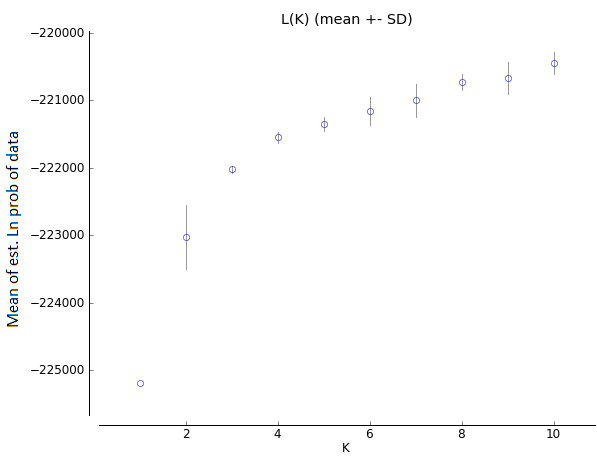 |
| --- | --- |

**Figure S2.** Graphical method (as in Evanno et al. 2005) allowing for detection of the number of groups K using (A) ΔK and (B) the rate of change of the likelihood distribution (mean log-likelihood values).


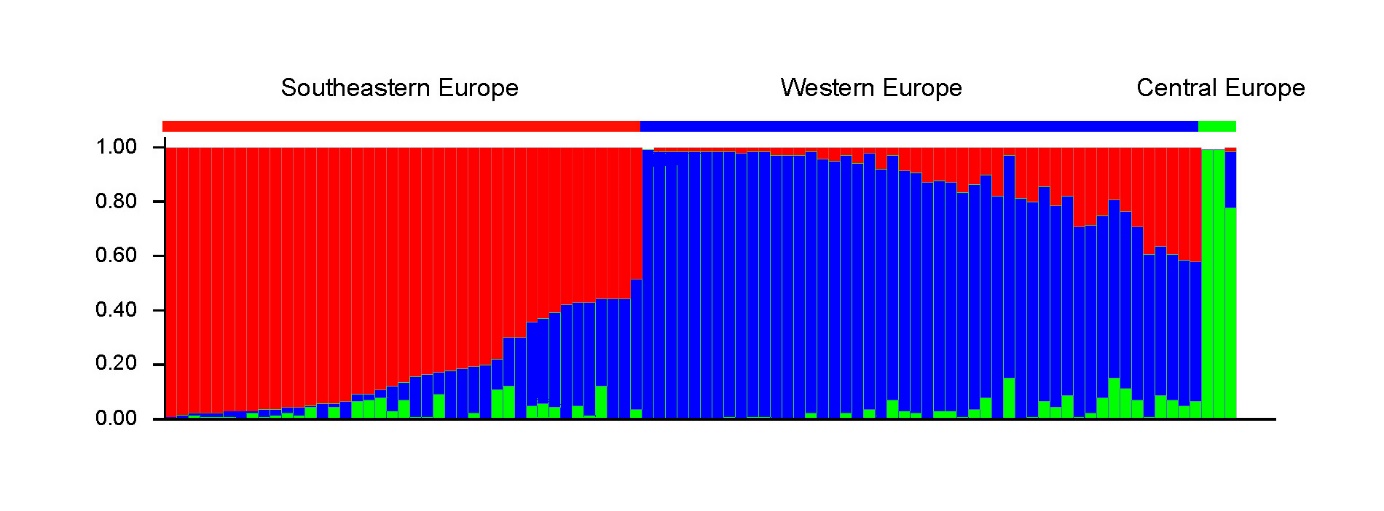


**Figure S3.** Bar plot of admixture proportions of individuals, inferred using K = 3 based on 2909 SNP loci. Individual’s proportions (*q*-values) are sorted within each cluster (cluster K1 – red, K2 – blue, K3 - green.


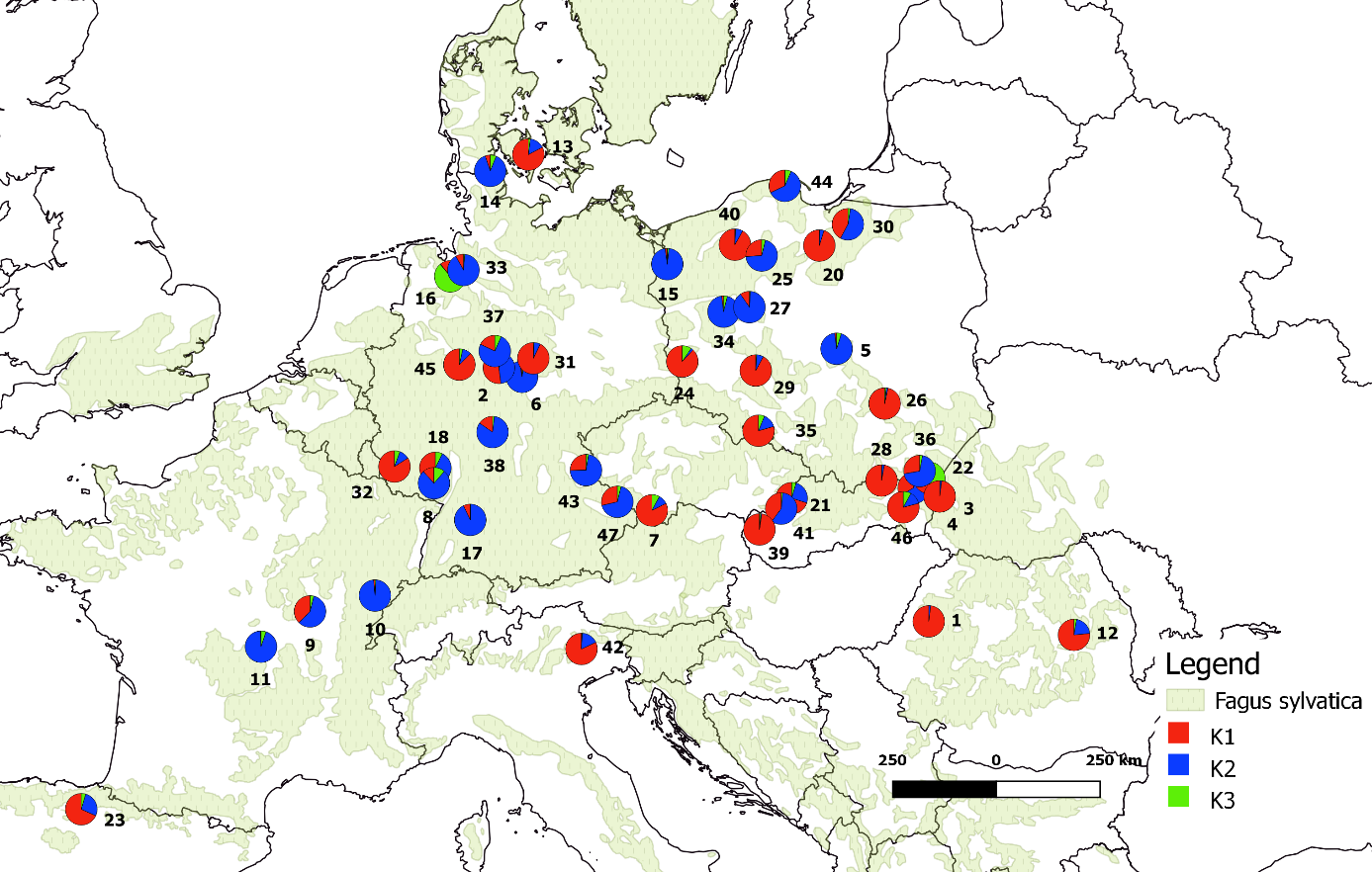


**Figure S4.** Geographical distribution of European beech clusters according to the K = 3 model in STRUCTURE.

| 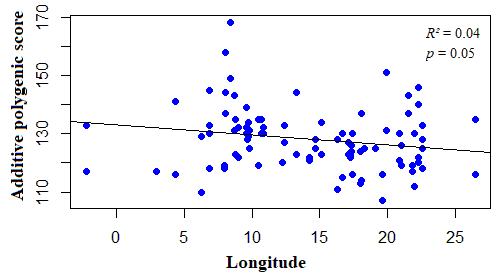 | 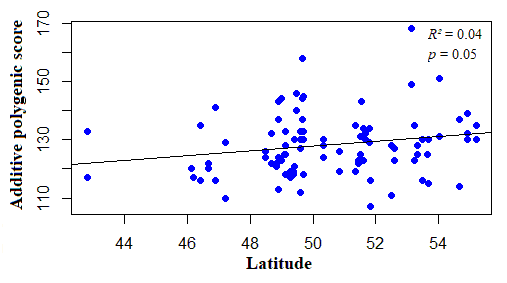 |
| --- | --- |
| 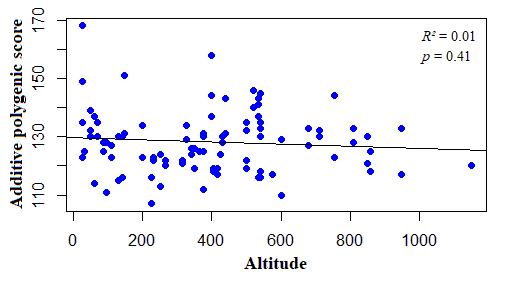 | 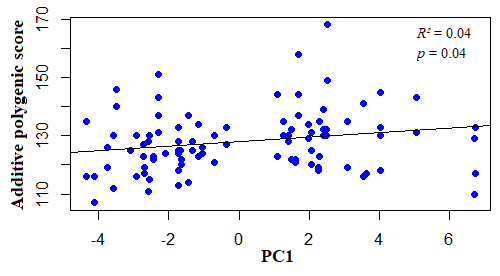 |
| 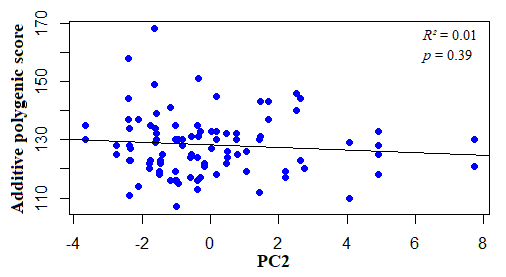 | 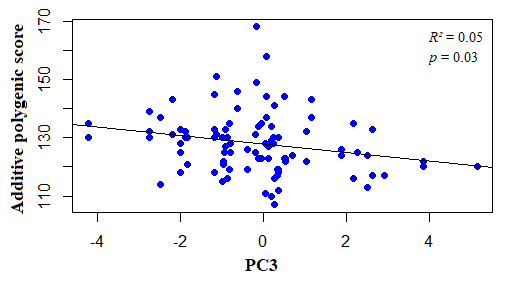 |
| 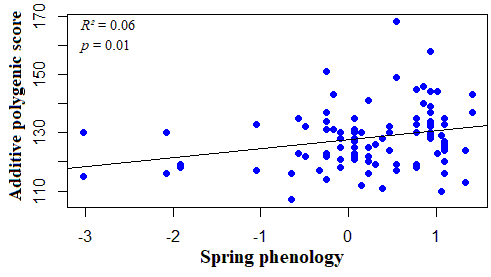 | 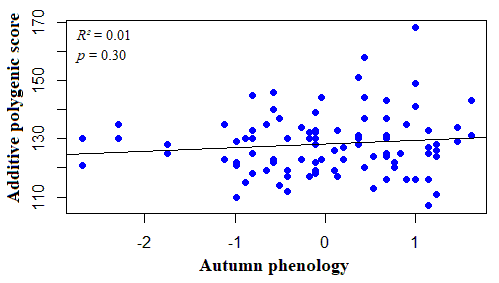 |
| 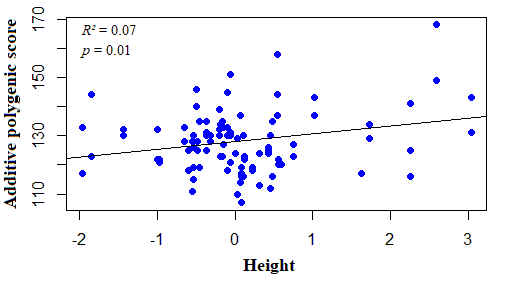 | 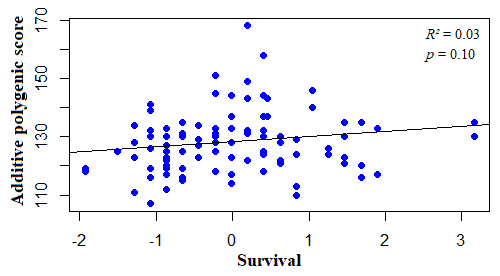 |

**Figure S5.** Relationships between additive individual polygenic scores based on all 201 outlier markers and each of the explanatory variables: geographic (longitude, latitude, altitude), climate (PC1, PC2, PC3) and phenotypic (spring phenology, autumn phenology, height, survival). The solid line represents the regression line of the linear model.

| 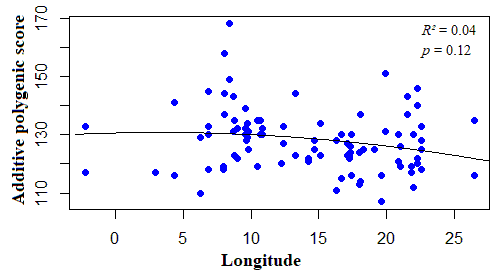 | 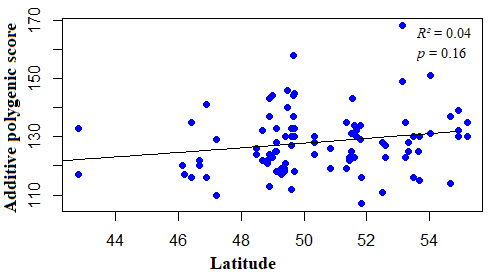 |
| --- | --- |
| 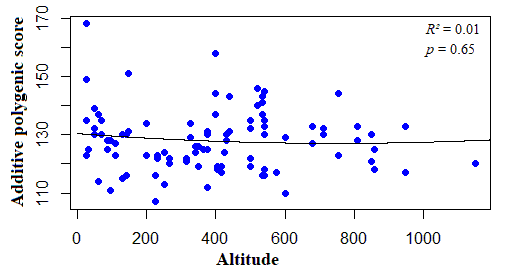 | 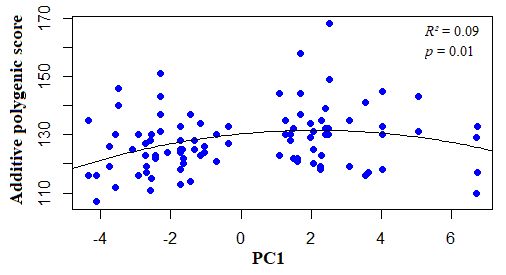 |
| 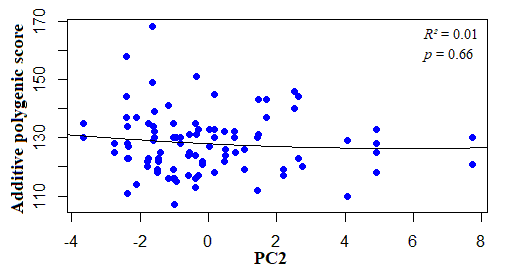 | 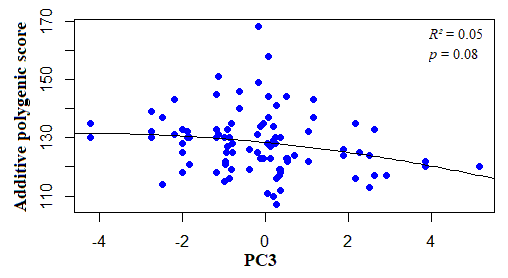 |
| 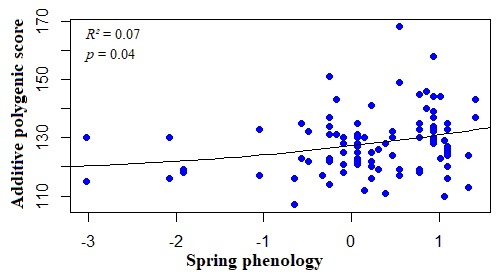 | 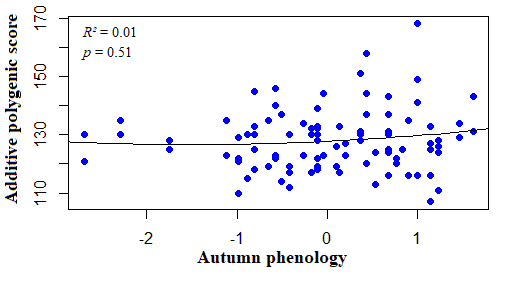 |
| 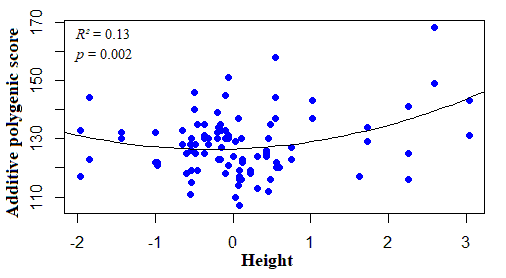 | 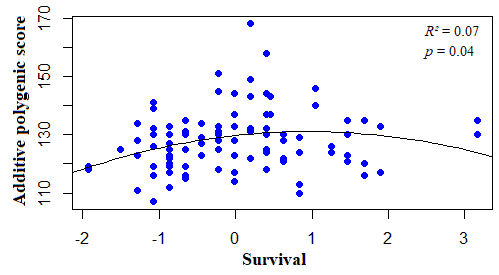 |

**Figure S6.** Relationships between additive individual polygenic scores based on all 201 outlier markers and each of the explanatory variables: geographic (longitude, latitude, altitude), climate (PC1, PC2, PC3) and phenotypic (spring phenology, autumn phenology, height, survival). The solid line represents the regression line of the quadratic model.
